# Conversion of a microwave synthesized alkali-metal MOF to a carbonaceous anode for Li-ion batteries†

**DOI:** 10.1039/d0ra01997f

**Published:** 2020-04-03

**Authors:** Aamod V. Desai, Vanessa Pimenta, Cara King, David B. Cordes, Alexandra M. Z. Slawin, Russell E. Morris, A. Robert Armstrong

**Affiliations:** School of Chemistry, East Chem, University of St. Andrews North Haugh, St. Andrews Fife KY16 9ST UK rem1@st-andrews.ac.uk ara@st-andrews.ac.uk; Department of Physical and Macromolecular Chemistry, Faculty of Science, Charles University Hlavova 8 128 43 Prague 2 Czech Republic

## Abstract

Hierarchical carbon-rich materials have shown immense potential for various electrochemical applications. Metal–organic frameworks (MOFs) are well suited precursors for obtaining such templated carbon matrices. Usually these conversions are carried out by energy intensive processes and lead to the presence of toxic transition metal residues. Herein, we demonstrate the green, scalable, microwave-assisted synthesis of a three-dimensional s-block metal based MOF and its efficient transformation into a carbonaceous material. The MOF-derived solid functions as a negative electrode for lithium-ion batteries having moderate low-rate capacities and cycling stability.

## Abstract 2

An alkali-metal MOF is prepared using microwave-assisted synthesis, which is converted into a carbonaceous solid at low energy costs. The MOF-derived solid functions as a promising anode for Li-ion rechargeable battery (LIB).

## Graphical abstract

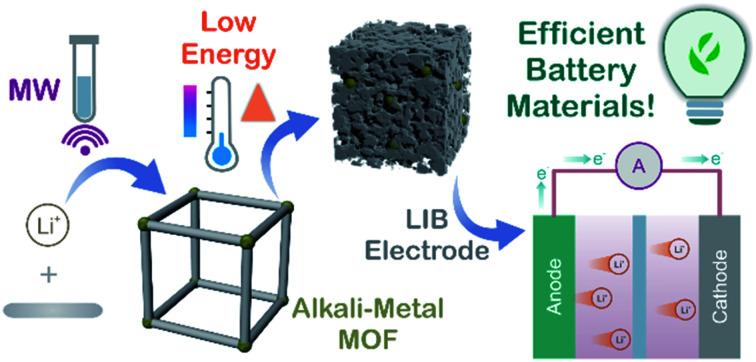

## Introduction

The continuously rising energy demands have actuated intense efforts in the domain of electrochemical energy storage (EES) technologies.^1–3^ Among other devices, rechargeable lithium-ion batteries (LIBs) have garnered remarkable research attention as they have been widely commercialized and are potentially suited for several applications.^4,5^ To comply with the widening scope, current research is focussed on the development of new materials for different battery components.^6^ For instance, carbon-rich materials are highly suited for negative electrodes, as evidenced by utility of graphite in commercialized LIBs. This has sparked interest in the exploration of diverse carbon-based and carbon-composite matrices for this application.^7,8^ The quest for electrode materials comprises of screening a range of practical parameters such as weight, cost, toxicity, energy density, cycling life *etc.* Hierarchical carbonaceous materials have demonstrated significantly superior performance over bulk materials, in addition to addressing several parameters described above.^9–12^ Thus research into deriving carbon from specific templates for battery electrodes is increasingly gaining greater prominence. Ordered, long range, crystalline solids are suitable precursors as they offer regularized surface area, which aids reversible ion insertion and provides space to absorb mechanical strain during the cycling process. As a rapidly emerging class of higher dimensional crystalline materials, metal–organic frameworks (MOFs) and their derivatives or composites have exhibited potential as functional materials for an array of electrochemical applications.^13–18^ The structural features of MOFs are carried further into their derivatives, making MOFs attractive self-sacrificial agents to cater for the demands of the desired electrochemical characteristics.^19–26^

Typically, MOF-derived carbons are prepared by the energy intensive process of pyrolysis at high temperatures under inert conditions. This process leads to homogeneous distribution of the metal nanoparticles or metal clusters embedded in the carbon matrix. Consequently, the choice of the building units has a direct influence on the derived matrix. Notably, very little research efforts have gone to investigate MOFs built from alkali or alkaline earth metals towards rechargeable battery electrodes, which are potentially useful on several counts.^27^ The large electronegativity differences in the bond forming atoms make the metal–carboxylate bond highly ionic in nature. The resulting frameworks deliver a density advantage over transition metal counterparts, and also score over them in terms of lower toxicity. It has been observed that predicting coordination geometries is not trivial in the case of s-block metals, and the resulting supramolecular assembly strongly depends upon the orientation of the functional groups.^27^ This permits examination of diverse coordination architectures by varying the building units and/or synthetic conditions. Particularly, metal–carboxylate assemblies of s-block elements have shown promise as anode materials,^28–31^ yet the research is significantly less developed compared to transition metal based solids or MOFs.

An important challenge in contemporary MOF research is the ability to synthesize on bulk scale, with energy efficient methods and creation of low-level waste.^32–34^ Among different approaches, microwave-assisted synthesis has gained remarkable interest owing to the ability to produce large scale compounds in substantially shortened reaction times, and its applicability to a wide range of solvents.^35,36^ The energy demands of synthesis and cost of precursors are vital parameters governing practical implementation. The cost of starting materials is the dominant contributor to the economic aspect of MOF synthesis.^37^ Furthermore the method of synthesis can significantly affect the energy costs and yields from a commercial perspective.^33^ In this regard, microwave-assisted preparation of active materials is particularly relevant to applications such as battery electrode materials.

**Fig. 1:**
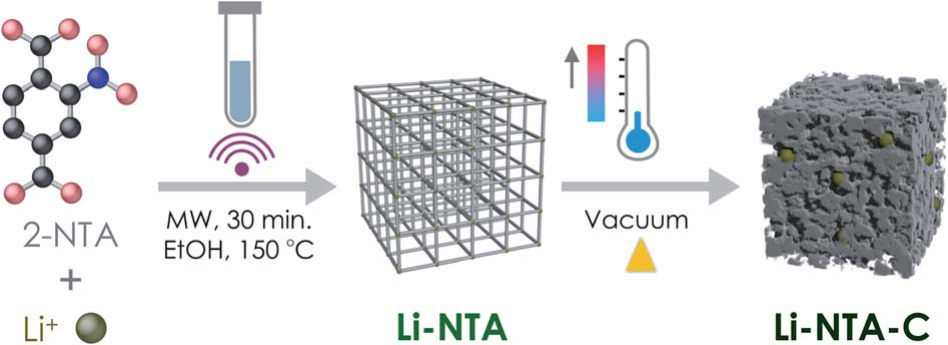
Schematic illustration of the synthetic protocol for Li-NTA and its derivative Li-NTA-C, towards anode material for Li-ion battery.

Based on this background and in our exploration of s-block MOFs for EES applications, we report the green, scalable microwave-assisted synthesis of a Li-based 3-dimensional MOF *viz.* Li-NTA (NTA stands for 2-nitro terephthalate), and its facile, energy-efficient conversion into hierarchical carbonaceous material (Li-NTA-C) (Fig. 1). The MOF-derived carbonaceous solid is promising as an anode for Li-ion batteries with significant low-rate capacities and cycling stability.

## Results and discussion

**Fig. 2:**
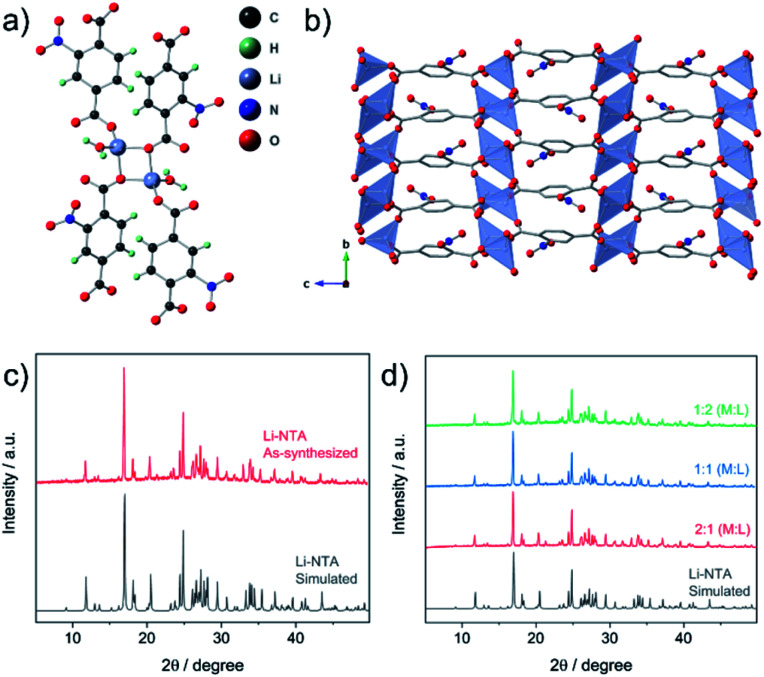
(a) Coordination environment in Li-NTA. (b) Packing diagram of Li-NTA along *a*-axis (H-atoms are omitted for clarity). (c) PXRD patterns for Li-NTA, simulated (grey) and as-synthesized phase (red). (d) PXRD patterns for compounds obtained by varying ratio of reagents (M: Li_2_CO_3_; L: 2-NTA).

The title compound (Li-NTA) was prepared using a microwave-assisted protocol, as described in the experimental section. The choice of the functionalized linker H_2_NTA was directed by the effect of pendant groups on coordination assembly of s-block networks^27^ and the role of N-doping in electrochemical activity.^20^ Single crystals of Li-NTA were obtained by slow evaporation of the crude product from water at room temperature. Neat colourless crystals were isolated and single-crystal X-ray diffraction (SC-XRD) analysis revealed the structure of Li-NTA. The compound crystallizes in the monoclinic space group *P*2_1_/*c*. The asymmetric unit comprises one ligand unit, two Li^+^ cations and two coordinated water molecules (Fig. S1 and S2†). Each Li^+^ has tetrahedral geometry, coordinating to 3 independent carboxylate ligands and one water molecule (Fig. 2a). The Li–O bond lengths vary from 1.89–1.99 Å, which is typically observed for tetrahedral lithium.^38^ The carboxylate moiety is bound to 3 independent Li^+^ cations, wherein the O1, O5 atoms have μ_2_-bridging binding mode. The packing is extended *via* one-dimensional extension of [Li_2_O_4_] units which cut through the *bc*-plane (Fig. S3†). The edges of every Li_2_O_4_ unit are capped by two water molecules. The overall structure gives rise to a stacked arrangement of the organic linker, each of which connect two adjacent, parallel [Li_2_O_4_] groups in the *bc*-plane (Fig. 2b). The pendant nitro (–NO_2_) groups have weak non-covalent interactions with the coordinated water molecule and adjoining linkers, which further strengthen the structural packing. Owing to contribution of such interactions, the framework has high density and the packing is tightly bound. The high density of the networks is particularly advantageous for certain electrochemical applications. The topology of the framework is *ant*, which is less frequently encountered in the literature of MOFs.^39^ Additionally, Li-NTA features among the uncommon class of 3-dimensional MOFs built from s-block elements.^40,41^ Li-NTA also benefits from the utilisation of relatively cheap precursors and adopting microwave synthesis as a sustainable approach.

**Fig. 3:**
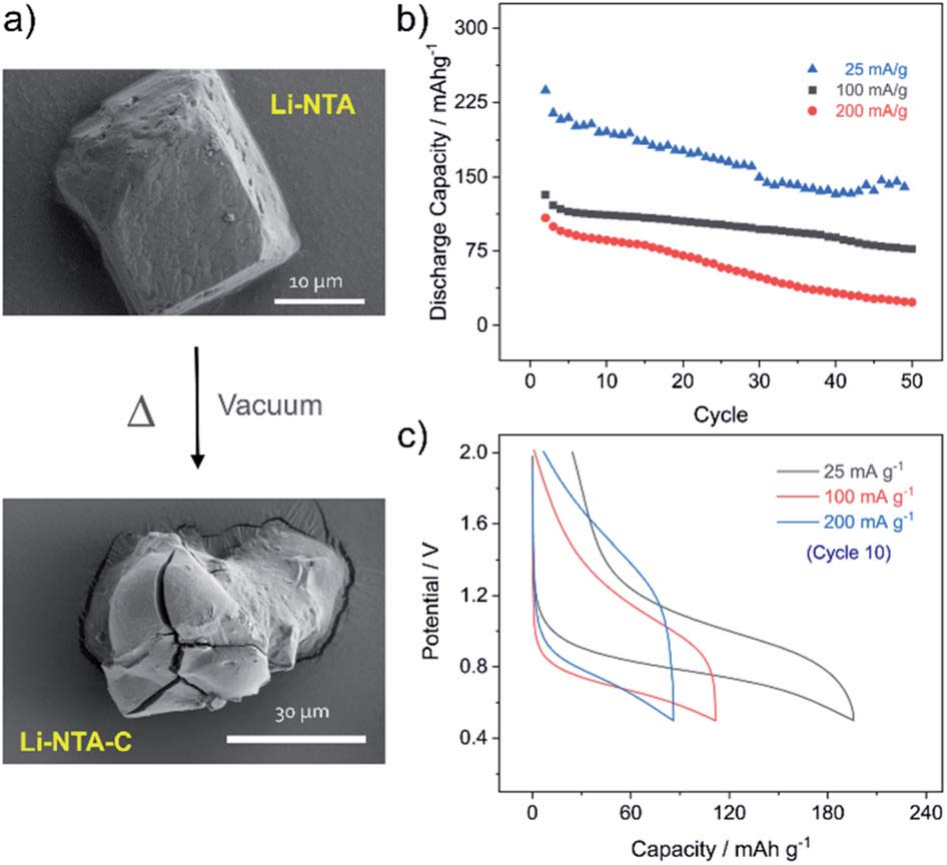
(a) FESEM images for pristine Li-NTA and Li-NTA-C. (b) Cycling data for Li-NTA-C from 0.5–2 V at different current densities. (c) Load curves for 10th cycle at rates of 25 mA g^−1^ (grey), 100 mA g^−1^ (red) and 200 mA g^−1^ (blue).

Powder X-ray diffraction (PXRD) patterns of the pristine phase were in good agreement with the simulated pattern, which validated the purity of the compound in the bulk phase (Fig. 2c). Fourier Transform Infrared (FT-IR) spectral analysis confirmed the presence of the peaks corresponding to carboxyl and the nitro groups (Fig. S9†). X-ray photon spectroscopy (XPS) patterns indicated presence of broad peak for O 1s, owing to contribution from multiple types of O-atoms present in the compound (Fig. S11†). The thermo-gravimetric analysis (TGA) profile displayed loss of coordinated water at ∼110 °C, after which a plateau was observed up to ∼380 °C with no further weight loss (Fig. S10†). This observation was supported by differential scanning calorimetry (DSC) profile (Fig. S10†), where a strong endothermic peak corresponding to the loss of the coordinated water molecule was observed. FESEM images of Li-NTA in the solid state revealed formation of block shaped morphology having agglomerated crystallites (Fig. 3a and S8†). To understand the structural formation of Li-NTA further, the synthesis was repeated by varying the molar ratios of the starting reagents (Fig. 2d). In all the cases the same compound was obtained with no evidence for formation of any side-products. The SEM analysis for the products obtained from varying concentrations endorsed the formation of similar shaped morphologies; although in the case where the ligand was in excess, the size of the crystallites was relatively larger (Fig. S5†). Li-NTA could be recrystallized in water and the same phase was obtained (Fig. S4†), which highlighted the utility of having purely ionic character^42^ in the coordination-bonded network.

As Li-NTA is composed of coordinated water molecules, further studies were carried out to understand the effect of water desorption on the structure. This study was particularly prompted by the structural characteristics of Li-NTA. As the coordination geometry around the hard Lewis acid Li(i) is tetrahedral, any loss of coordinating groups would destabilize the local environment. Variable temperature PXRD (VT-PXRD) profiles were recorded under vacuum and in air up to 340 °C (Fig. S6 and S7†). The loss of coordinated water accompanied by structural change was evident in the VT-PXRD patterns with diminishing low-angle peaks. Similar patterns were observed for the sample heated under vacuum, but the loss of water occurred at a lower temperature. The compound recovered from the heating experiment had drastic change to the colour from white crystalline to black powder. Seemingly, the loss of coordinated water molecule results in the decomposition of the crystalline structure^43^ and leads to formation of carbonaceous matter (hereafter referred as Li-NTA-C). The SEM images for Li-NTA-C suggested a transition from well-defined morphology to softening of the edges and amorphous character (under air), and complete transformation into amorphous solid (under vacuum) with no defined features (Fig. 3a and S8†). FTIR spectra for Li-NTA-C showed the absence of the carboxyl peaks, which further suggested transformation of the pristine phase (Fig. S9†). XPS analysis confirmed the phase transition and relatively sharper peaks for O 1s at higher binding energy and similarly for C 1s, and retention of the peak for Li, suggested formation of Li-particles embedded within carbon matrix (Fig. S11†). Room temperature PXRD indicated formation of Li_2_CO_3_ with no competing peaks, which was also observed in the peak for carbonate stretching in the FT-IR spectrum (Fig. S9 and S12b†). This observation can be supported by the purely ionic character of the coordination bond in Li-NTA and the known decomposition of terephthalic acid at elevated temperatures.^44^ The presence of a dissociating group facilitates the disruption of the coordination network and leads to decomposition into carbonaceous material. Thus, Li-NTA-C presents an energy efficient approach to obtain ordered carbonaceous matrices from higher dimensional supramolecular architectures of purely ionic solids. A systematic study on these lines can further provide insight into the role of molecular topology in MOF-derived carbonaceous materials.

Subsequently, Li-NTA-C was explored as an anode material for Li-ion batteries. The electrochemical studies were performed by mixing pristine phase of Li-NTA with conductive carbon (Super C65) and carboxymethyl cellulose (CMC) as the binder in the ratio of 65 : 25 : 10. The electrode slurry was prepared in water and dried under vacuum at 110 °C for 12 h, to attain the carbonaceous material (Li-NTA-C) directly in the electrode mix. The fabricated electrodes were then assembled into coin cells and cycling studies were carried out in the potential window of 0.5–2 V. At low current rate (25 mA g^−1^) the discharge potential was observed at ∼0.8 V, while the charge peak appeared at ∼1.0 V (Fig. S13†). A large irreversible capacity was observed for the first cycle, which could attributed to the formation of solid–electrolyte interface (SEI) and contribution of capacity from carbon additive.^45^ The cycling performance was subsequently recorded at different rates (25, 100, 200 mA g^−1^) (Fig. 3b). At rate of 25 mA g^−1^, an initial discharge capacity of ∼240 mA h g^−1^ was noted, which decreased gradually to ∼150 mA h g^−1^, after which a plateau was observed for more than 20 consecutive cycles. A much more stable cycling performance was observed at rate of 100 mA g^−1^, where the initial capacity of ∼140 mA h g^−1^ was stabilized over multiple cycles. Doubling the current rate did not significantly perturb the capacity for initial cycling, but for multiple cycles the capacity reduced. Load curves for the 10th cycle at different discharge rates are shown in Fig. 3c. The cycling stability was found to be maintained, with the low rate capacities recovered after cycling at 400 mA g^−1^ (Fig. S14†). Apart from acceptable capacity and cycling performance, Li-NTA-C benefits from significantly lower toxicity of metals involved, synthetic ease and cost considerations.^46–49^ Commercial graphite anode materials have a number of limitations, particularly with regard to safety, stimulating development of novel systems.^50^ Hierarchical carbon materials have been found to overcome certain limitations; Li-NTA-C contributes to these materials by virtue of relatively stable low-rate capacities and safer working voltage. Further, this approach of converting thermally less stable, higher dimensional MOFs may lead to exploring potential of MOF-derivatives prepared at lower costs by energy efficient processes.

## Conclusions

In summary, the facile synthesis of s-block based functional MOF (Li-NTA) and its energy efficient transformation into a carbonaceous material is reported. The parent MOF is prepared utilizing the green, scalable approach of microwave-assisted synthesis. Li-NTA undergoes irreversible and energy efficient structural transformation upon heating to form a carbonaceous solid (Li-NTA-C). Li-NTA-C exhibits promise as anode material for Li-ion batteries with moderate capacities at low discharges rates and cycling stability. The present findings contribute to the development of MOFs based on alkali metal ions, and MOF-derived materials for electrochemical applications, especially for rechargeable batteries.

## Conflicts of interest

There are no conflicts to declare.

## Supplementary Material

RA-010-D0RA01997F-s001
