# Supplementary material for: Conversion of a microwave synthesized alkali-metal MOF to a carbonaceous anode for Li-ion batteries
Source: RSC Adv. 2020 Apr 3;10(23):13732–6. doi: 10.1039/d0ra01997f (PMC9051547; doi:10.1039/d0ra01997f)
Supplement: RA-010-D0RA01997F-s001 [file RA-010-D0RA01997F-s001.pdf]

Electronic Supplementary Material (ESI) for RSC Advances.
This journal is © The Royal Society of Chemistry 2020

Supporting Information

Conversion of a Microwave Synthesized Alkali-Metal MOF to Carbonaceous Anode for Li-Ion Batteries

Aamod V. Desai,[a] Vanessa Pimenta,[a] Cara King,[a] David B. Cordes,[a] Alexandra M. Z. Slawin,[a] Russell E. Morris[a,b,*] and A. Robert Armstrong[a,*]

[a] School of Chemistry, East Chem, University of St. Andrews., North Haugh, St. Andrews, Fife, KY16 9ST, United Kingdom.

[b] Department of Physical and Macromolecular Chemistry, Faculty of Science, Charles University, Hlavova 8, 128 43, Prague 2, Czech Republic.

* Email: rem1@st-andrews.ac.uk ; ara@st-andrews.ac.uk

Experimental Details

Synthesis of Li-NTA: The ligand 2-nitroterephthalic acid [H_2NTA] (264 mg, 1.25 mmol) and metal salt - lithium carbonate (92.5 mg, 1.25 mmol) were added directly as solid to the microwave reaction tube. To this mixture ethanol (4 ml) was added and the tube was sealed. The mixture was then allowed to react under Microwave irradiation at 423 K for 30 minutes, after 30 minutes of pre-heat stirring. Upon cooling a solid precipitate was obtained which was washed several times with ethanol. This product was then dissolved in deionized water (~4 ml) and filtered. The filtrate obtained was left for drying in oven. Upon complete drying, a white solid was obtained in ~65% yield. For obtaining single-crystals the dissolved product was left standing for evaporation at room temperature. Anal. calcd. for Li-NTA $\{[Li_2(NTA)(H_2O)_2\}_n$: C, 37.10; H, 2.72; N, 5.41. Found: C, 36.97; H, 2.58; N, 5.32. The protocol and amount of ligand was retained for ratio-variable synthesis. Elemental analysis for Li-NTA-C was found to have the following composition: C, 36.12; H, 2.68; N, 4.59.

Structural Characterization: X-ray diffraction data for Li-NTA were collected at 125 K using a Rigaku MM-007HF High Brilliance RA generator/confocal optics with XtaLAB P200 diffractometer [Cu Kα radiation (λ = 1.54187 Å)]. Intensity data were collected using ω steps accumulating area detector images spanning at least a hemisphere of reciprocal space. Data were collected using CrystalClear[1] and processed (including correction for Lorentz, polarization and absorption) using CrysAlisPro.[2] Structures were solved by direct methods (SIR2011[3]) and refined by full-matrix least-squares against F^2 (SHELXL-2018/3[4]). Non-hydrogen atoms were refined anisotropically, and aromatic hydrogen atoms were refined using a riding model. Hydrogen atoms on water were located from the difference Fourier map and refined isotropically subject to a distance restraint. All calculations were performed using the CrystalStructure[5] interface. Selected crystallographic data are presented in Table S1. CCDC 1971826 contains the supplementary crystallographic data for this paper. The data can be obtained free of charge from The Cambridge Crystallographic Data Centre via www.ccdc.cam.ac.uk/structures. Powder X-ray diffraction (PXRD) patterns were recorded on a STOE STADI/P diffractometer using Cu $K_{\alpha 1}$ radiation in glass capillaries at 298 K. Variable temperature PXRD was collected under vacuum on a PANalytical Empyrean X'Celerator RTMS detector diffractometer using Mo $K_{\alpha 1,2}$ radiation in an alumina sample holder. Thermogravimetric analysis (TGA) was performed in air, using a Netzsch thermogravimetric analyzer TG 209 from ambient temperature to 700 °C, with a 10 °C per minute heating rate. Differential scanning calorimetry (DSC) data was obtained on Netzsch DSC 204 F1 Phoenix for heating cycle up to 300 °C at a heating rate of 10 °C/min under N_2 atmosphere. SEM

images were recorded using a Jeol JSM 5600 SEM after Au-coating the samples. IR spectroscopy was performed using a Shimadzu IR affinity-1 FTIR spectrophotometer in the range of 400-4000 cm^{-1}.

Electrochemical Characterization: The working electrode was prepared by mixing the active material (Li-NTA) with conductive carbon (Super P) and binder (CMC) in water as the solvent. The ratio was maintained as 65:25:10 respectively. The prepared slurry was then cast on Aluminium foil using a doctor blade and dried overnight. The approximate active mass loading per disc was ~1 mg cm^{-1}. Electrodes were incorporated into coin cells (type CR2325, NRC Canada) with Lithium metal as counter electrode and LP 30 (Sigma-Aldrich) as the electrolyte. The cells were assembled in an argon-filled glovebox (MBraun) with moisture content and oxygen levels below 1 ppm. The electrochemical measurements were performed at room temperature using a Biologic MacPile II system, while waterfall plot was obtained at 30 °C on a Maccor Series 4200 battery cycler.

Figures

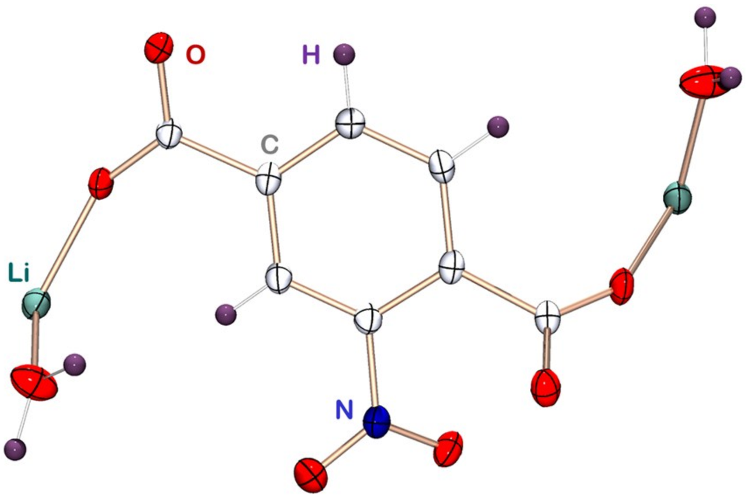

Figure S1. ORTEP[6-7] diagram for asymmetric unit of Li-NTA. Ellipsoids are drawn at 50% probability level.

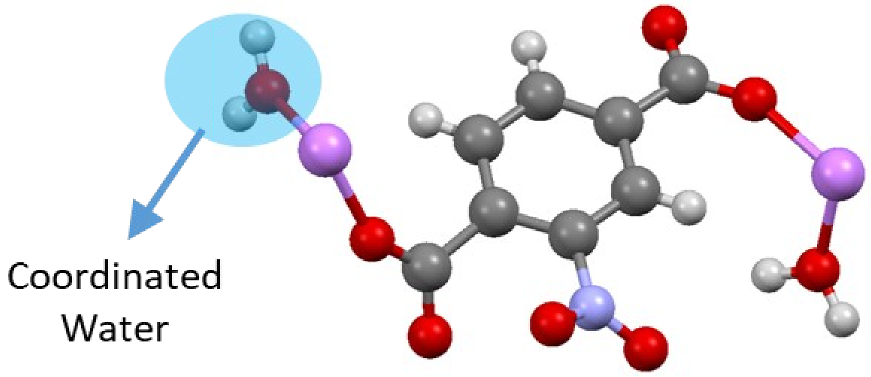

Figure S2. Asymmetric unit of Li-NTA showing coordinated water to Li-center. (C, grey; N, blue; O, red; Li, pink; H, light grey).

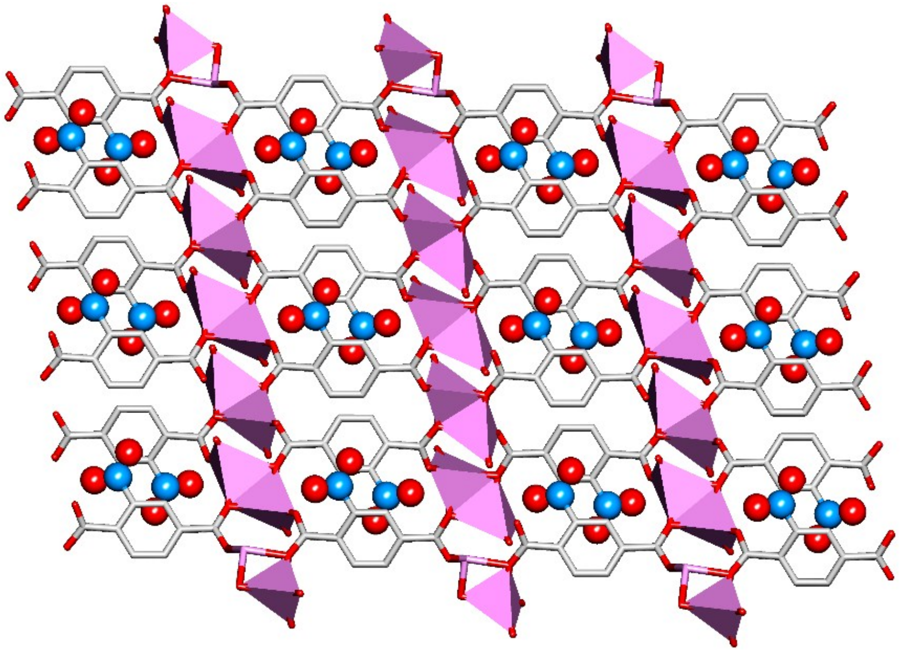

Figure S3. Packing diagram for Li-NTA. (C, grey; N, blue; O, red; Li, pink. H-atoms omitted for clarity)

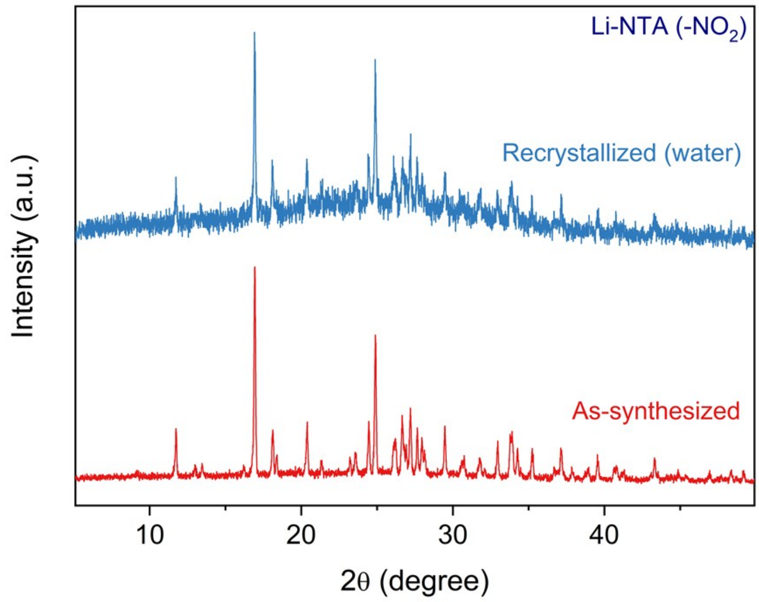

Figure S4. Comparative PXRD patterns for as-synthesized powder (red) and recrystallized phase from water (blue).

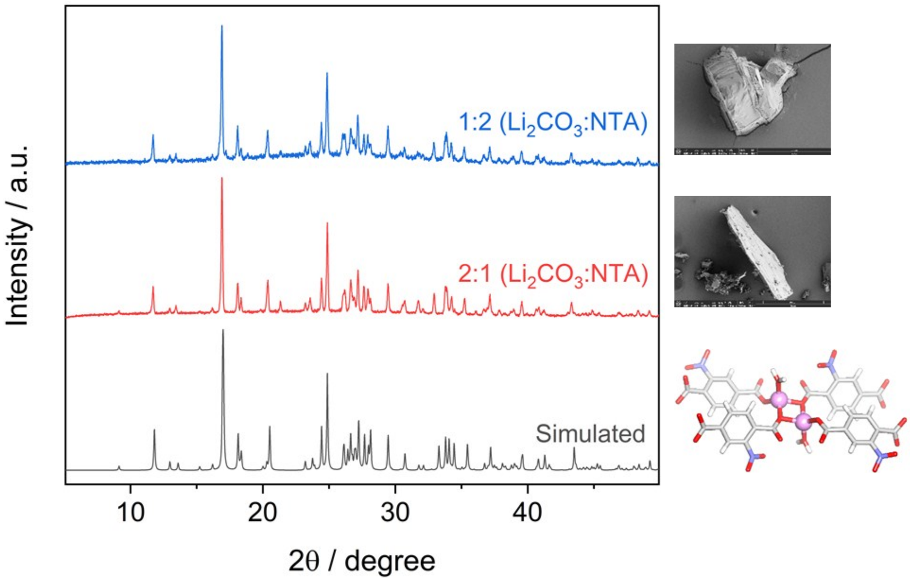

Figure S5. PXRD patterns for Li-NTA obtained from different molar ratios of the starting materials [Metal salt/Ligand: 2/1 (red); 1/2(blue)]. Corresponding FESEM images are shown alongside.

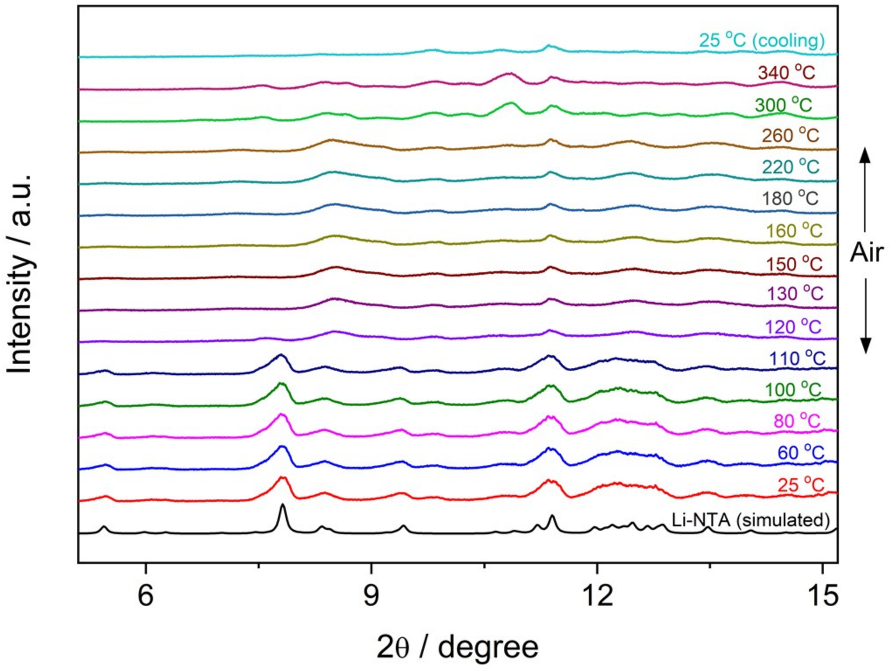

Figure S6. Variable temperature PXRD (VT-PXRD) patterns recorded in air.

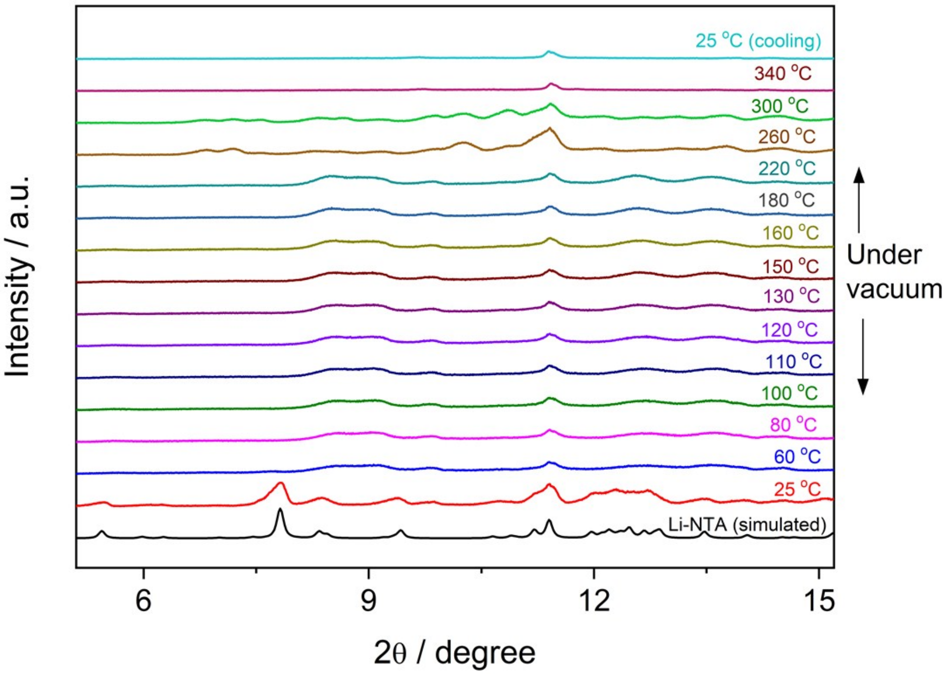

Figure S7. Variable temperature PXRD (VT-PXRD) patterns recorded under vacuum.

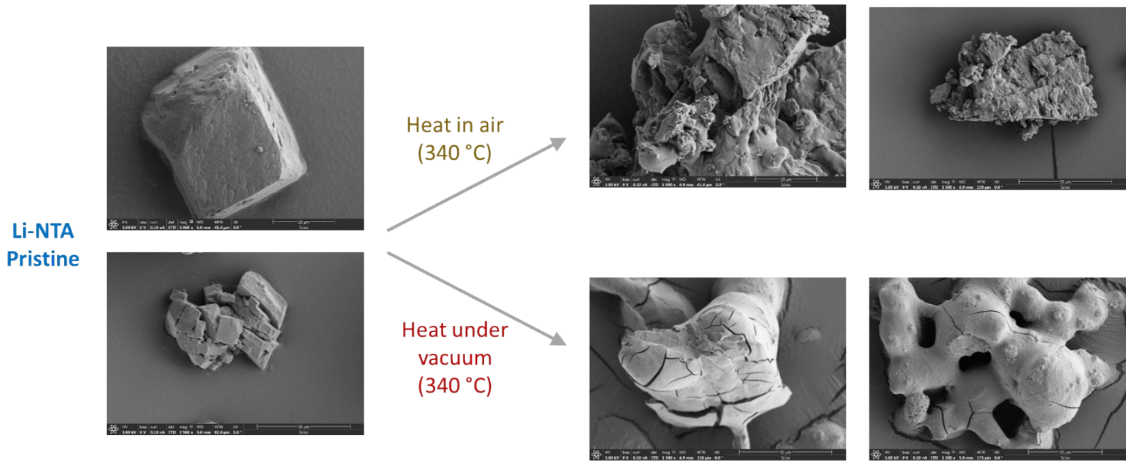

Figure S8. FESEM images for the pristine Li-NTA (left) and the phases obtained after heating at 340 °C, in air (top) and under vacuum (below).

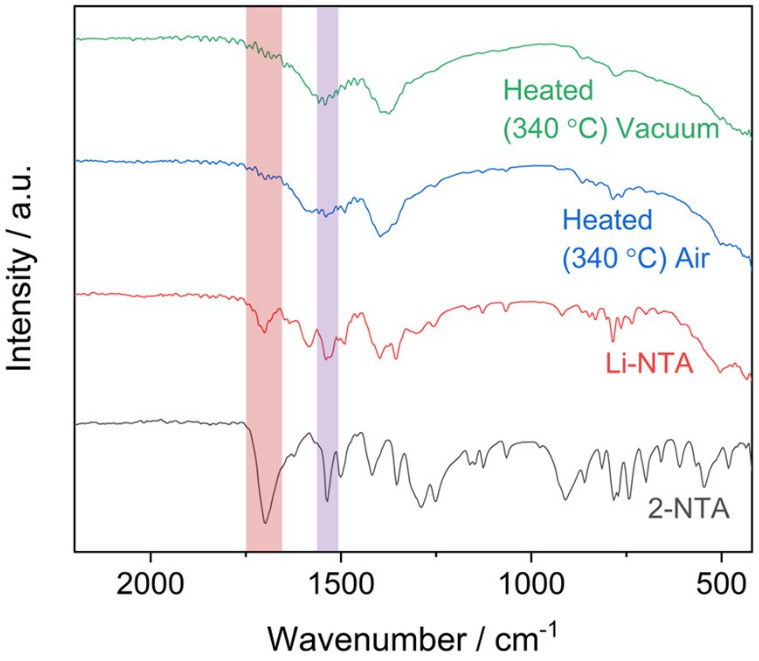

Figure S9. FT-IR spectra for ligand (grey), Li-NTA (red), and the phases obtained after heating at 340 °C, in air (blue) and under vacuum (green). The peaks corresponding to the carboxylate (red) and nitro (purple) groups are highlighted.

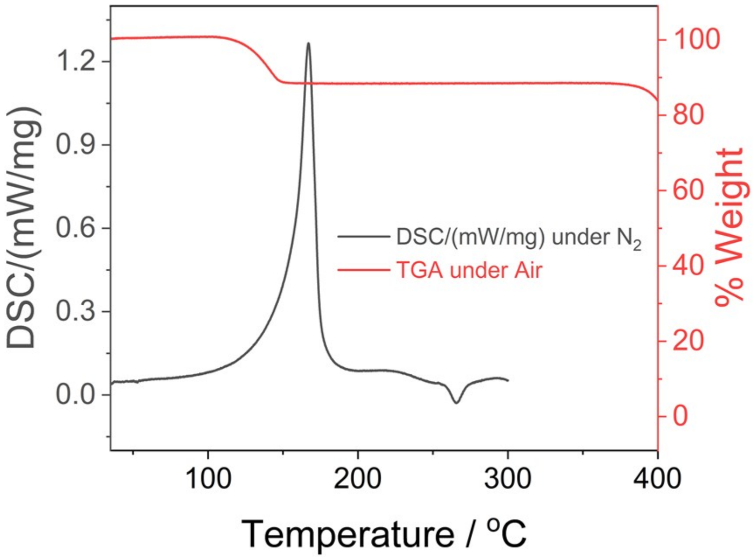

Figure S10. TGA (red) and DSC profile (grey) for Li-NTA.

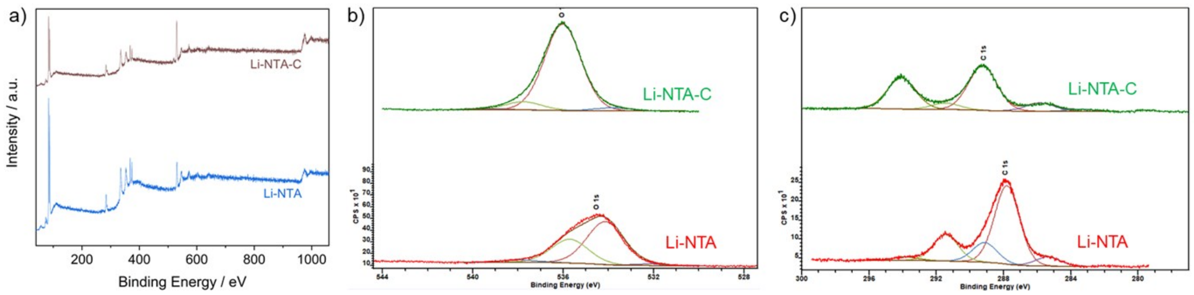

Figure S11. a) Stacked plot of XPS survey scan for Li-NTA and Li-NTA-C. Comparative spectra for b) O1s and c) C1s signal.

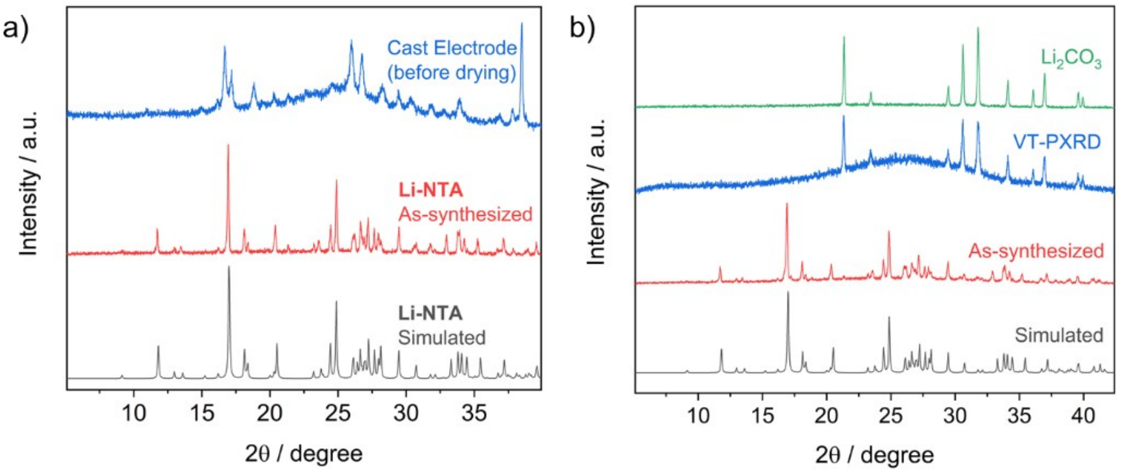

Figure S12. a) PXRD patterns for the cast electrode phase (blue) and comparison with as-synthesized and simulated patterns. b) PXRD patterns for compound recovered from VT-PXRD experiment (blue) and for Li_2CO_3 (green).

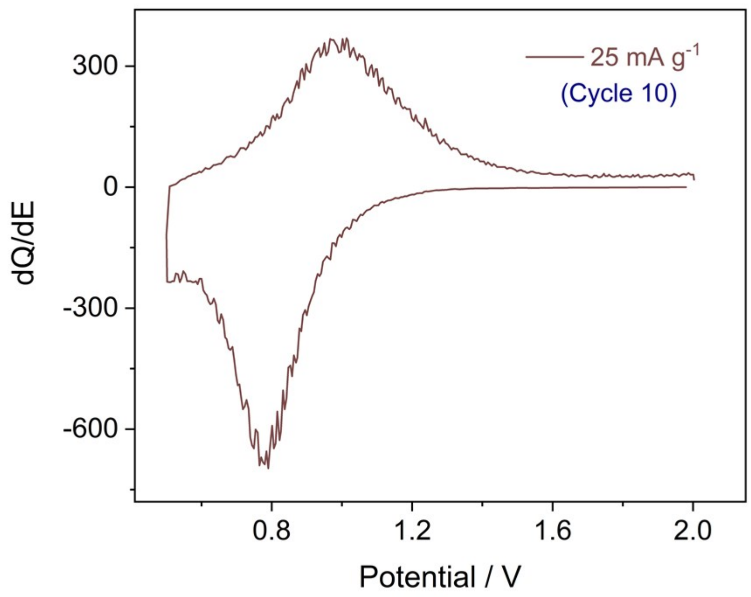

Figure S13. Plot of dQ/dE vs potential for 10th cycle at current rate of 25 mA/g.

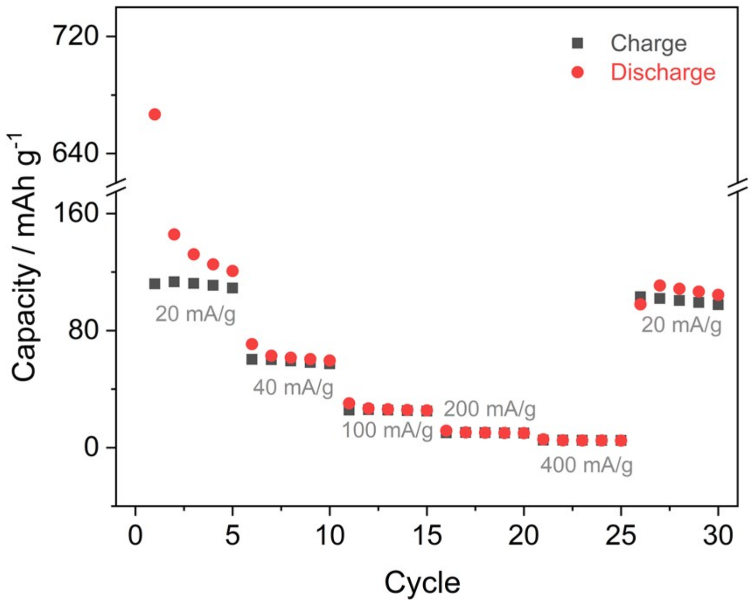

Figure 14. Rate performance for Li-NTA between 0.5-2.0 V at different rates (shown alongside respective dataset).

Table S1. Selected crystallographic data.

	**Li-NTA**
empirical formula	$C_8H_7Li_2NO_8$
fw	259.03
crystal description	colourless chip
crystal size [mm^3]	0.08×0.06×0.02
space group	$P2_1/c$
a [Å]	7.60716(15)
b [Å]	7.27980(14)
c [Å]	19.6363(4)
β [°]	100.2380(19)
vol [Å]^3	1070.12(4)
Z	4
ρ (calc) [g/cm^3]	1.608
μ [mm^{-1}]	1.253
F(000)	528
reflections collected	12376
independent reflections (R_{int})	2178 (0.0311)
data/restraints/parameters	2178/4/188
GOF on F^2	1.085
R_1 [$I > 2\sigma(I)$]	0.0385
wR_2 (all data)	0.1034
largest diff. peak/hole [e/Å^3]	0.38, -0.29

References:

1. *CrystalClear-SM Exper*t v2.1. Rigaku Americas, The Woodlands, Texas, USA, and Rigaku Corporation, Tokyo, Japan, 2015.

2. *CrysAlisPro* v1.171.39.8d. Rigaku Oxford Diffraction, Rigaku Corporation, Oxford, U.K. 2015.

3. M. C. Burla, R. Caliandro, M. Camalli, B. Carrozzini, G. L. Cascarano, C. Giacovazzo, M. Mallamo, A. Mazzone, G. Polidori and R. Spagna, R. *J. Appl. Cryst*., 2012, **45**, 357-361.

4. G. M. Sheldrick, *Acta Crystallogr., Sect. C*., 2015, **71**, 3-8.

5. *CrystalStructure* v4.3.0. Rigaku Americas, The Woodlands, Texas, USA, and Rigaku Corporation, Tokyo, Japan, 2018.

6. M. N. Burnett and C. K. Johnson, *ORTEP-III*: Oak Ridge Thermal Ellipsoid Plot Program for Crystal Structure Illustrations, Oak Ridge National Laboratory Report ORNL-6895, 1996.

7. L. J. Farrugia, *J. Appl. Cryst.,* 2012, **45**, 849-854.
